# Relative Fitness of Fluoroquinolone-resistant *Streptococcus pneumoniae*

**DOI:** 10.3201/eid1106.040840

**Published:** 2005-06

**Authors:** Crystal N. Johnson, David E. Briles, William H. Benjamin, Susan K. Hollingshead, Ken B. Waites

**Affiliations:** * University of Alabama at Birmingham, Birmingham, Alabama, USA

**Keywords:** animal model, antimicrobial resistance, fluoroquinolone, nasopharyngeal carriage, pneumonia, streptococcus pneumoniae, fitness

## Abstract

Fluoroquinolone resistance in *Streptococcus pneumoniae* is primarily mediated by point mutations in the quinolone resistance–determining regions of *gyrA* and *parC*. Antimicrobial resistance mutations in housekeeping genes often decrease fitness of microorganisms. To investigate the fitness of quinolone-resistant *S. pneumoniae* (QRSP), the relative growth efficiencies of 2 isogenic QRSP double mutants were compared with that of their fluoroquinolone-susceptible parent, EF3030, by using murine nasopharyngeal colonization and pneumonia models. Strains containing the GyrA: Ser81Phe, ParC: Ser79Phe double mutations, which are frequently seen in clinical QRSP, competed poorly with EF3030 in competitive colonization or competitive lung infections. However, they efficiently produced lung infection even in the absence of EF3030. The strain containing the GyrA: Ser81Phe, ParC: Ser79Tyr double mutations, which is seen more frequently in laboratory-derived QRSP than in clinical QRSP, demonstrated reduced nasal colonization in competitive or noncompetitive lung infections. However, the strain was equally able to cause competitive or noncompetitive lung infections as well as EF3030.

*Streptococcus pneumoniae* causes otitis media, bacteremia, and meningitis and is a leading cause of community-acquired bacterial pneumonia worldwide. Pneumococcal infections are commonly treated with β-lactams, macrolides, and, increasingly, fluoroquinolones. Pneumococcal resistance to each of these drug classes has increased in recent years (1,2). Initially, antimicrobial resistance in a pathogen may come at a cost: modifications that allow survival in the presence of antimicrobial drugs may render the pathogen less efficient at host infection, even in the absence of the antimicrobial agent (3). Little is known about the fitness of antimicrobial-resistant *S. pneumoniae* (4–8). The emergence of quinolone-resistant *S. pneumoniae* (QRSP) appears to be more dependent on fluoroquinolone selection of de novo spontaneous point mutations in the quinolone resistance–determining regions (QRDRs) of the topoisomerase genes *gyrA* and *parC* than on clonal dissemination (9–13). However, some studies reported occurrences of clonal relatedness among QRSP (11,14–16).

To investigate the relative fitness of QRSP, we conducted a competition study of a fluoroquinolone-susceptible clinical strain of *S. pneumoniae* (EF3030) with 2 of its fluoroquinolone-resistant isogenic mutants that had 2 common QRDR point mutation combinations. These 3 strains were analyzed by using an in vitro growth model, an in vivo nasopharyngeal colonization model, and an in vivo pneumonia model. We also carried out the nasopharyngeal colonization and pneumonia infections in the absence of competition to assess the ability of the mutants to colonize and to produce pneumonia in the absence of competition from the susceptible parent. To our knowledge, this is the first extensive investigation into the relative fitness of QRSP using in vitro models in combination with nasopharyngeal colonization and lung infection models.

## Materials and Methods

### Generation of Fluoroquinolone-resistant Mutants

For this study, naturally occurring fluoroquinolone resistance mutations were placed in the serotype 19F strain EF3030 by using established techniques (17). Briefly, 1,325-bp fragments of *gyrA* and 778 bp of *parC* were amplified by polymerase chain reaction from 2 previously characterized (18) clinical isolates of QRSP (CT01147 and UAB169; gemifloxacin MIC = 1 μg/mL) by using the primers shown in the Table. Phenotypic expression was carried out for 2 to 24 h. First-step transformants were generated by the introduction of a *parC* or *gyrA* fragment, and these were selected on 0.06 μg/mL gemifloxacin (SmithKline Beecham Pharmaceuticals, Collegeville, PA, USA). Second-step transformants were generated by the introduction of the second fragment (*gyrA* or *parC*) into first-step transformants, and these were selected on 0.5 μg/mL gemifloxacin, a concentration that effectively inhibited the growth of first-step mutants and permitted the growth of second-step mutants. Two of the isogenic *gyrA*, *parC* double mutants, Phe/Phe and Phe/Tyr, were chosen for fitness studies. In addition, levofloxacin MICs were determined for these 2 mutants by using broth microdilution.

**Table:** Primers used in the study

Name	Sequence	Product size (bp)
gyrA-F	5´-TTTAGGTGAAGTGAAGGCAAGGG-3´	1,325
gyrA-R	5´-GAATAACATTGGCTGAGGCGTC-3´	
parC-F	5´-TTTGAAAGGAGTTGAACACGCC-3´	778
parC-R	5´-TCCGTCCATAGAACCGTTATTACC-3´	

### Competitive Growth of QRSP Mutants In Vitro

Phe/Phe contained a GyrA: Ser81Phe mutation and a ParC: Ser79Phe mutation. Phe/Tyr contained a GyrA: Ser81Phe mutation and a ParC: Ser79Tyr mutation. In vitro competition experiments were carried out between EF3030 and Phe/Phe (N = 7) and between EF3030 and Phe/Tyr (N = 12) by coincubating them in Todd-Hewitt broth containing yeast extract (Difco, Detroit, MI, USA). The number of generations of each strain was calculated as previously described (4) by using the formula g = (logB – logA)/(log2), where relative fitness (RF) = g_res_/g_sus_, g is the number of generations, res is gemifloxacin-resistant transformants (Phe/Phe or Phe/Tyr), sus is the gemifloxacin-susceptible parent EF3030, B is the CFU/mL at time 1 (6 h), and A is the CFU/mL at time 0.

### Murine Pneumonia Models

For both models of pneumococcal infection, 6-week-old, female CBA/CaHN-*Btk*^xid^/J (CBA/N) mice (Jackson Laboratories, Bar Harbor, ME, USA) were used. Infection leading to pneumonia and colonization were induced over a period of 7 days, and samples were obtained from nasopharynges, lungs, and blood of mice as previously described (19,20). The pneumonia model entailed anesthetizing the mouse by inhalation of isoflurane before delivery of bacteria in 40 μL of lactated Ringer solution to ensure delivery to the lungs. In the colonization model, nonanesthetized mice were infected intranasally with bacteria in 10μL of lactated Ringer solution to ensure colonization of the nasopharynx, as previously described (19,20). All mouse experiments were carried out under the approval of the Institutional Animal Care and Use Committee at the University of Alabama at Birmingham.

### Competitive Growth of QRSP Mutants In Vivo

To determine relative nasopharyngeal growth during colonization, 10 μL of a 1:1 mixture containing 10^6^ CFUs each of EF3030 and the fluoroquinolone-resistant mutant (Phe/Phe or Phe/Tyr) were instilled into the nares as described for the colonization model (20). Ten mice received the EF3030 and Phe/Phe mixture, and 10 mice received the EF3030 and Phe/Tyr mixture.

To determine relative growth in the lungs, 40 μL of a 1:1 mixture containing EF3030 and the fluoroquinolone-resistant mutant (Phe/Phe or Phe/Tyr) were instilled into the nares as previously described for the pneumonia model (19). For the EF3030 and Phe/Phe competitive infections, 9 mice received 10^4^ CFUs of each strain, and 14 mice received 10^6^ CFUs of each strain. For the EF3030 and Phe/Tyr competitive infections, 9 mice received 10^4^ CFUs of each strain, and 14 mice received 10^6^ CFUs of each strain. Initially, the lower dose was used because of concerns for mouse mortality. When this turned out not to be an issue, the infectious dose was raised to 10^6^ CFU to increase lung infection levels, yield more countable colonies, and allow the effects of a range of infectious doses to be examined.

For recovery of EF3030 and mutants from mice in the pneumonia and colonization models, mice were killed 7 days postinfection, samples were collected, and CFUs were counted in nasal washes, lungs, and blood as described previously (19,20). Serial dilutions of specimens were cultured with gentamicin (which allowed growth of EF3030 and both mutants but reduced growth of oral commensal organisms) and with gemifloxacin (which allowed growth of only Phe/Phe and Phe/Tyr). Samples were incubated on blood agar plates containing 5 μg/mL gentamicin with or without 0.08 μg/mL gemifloxacin at 37°C for 16 h in a candle jar.

Percentage recovery units (PRUs) were determined for bacteria recovered from mice co-colonized or coinfected with both strains. PRUs were calculated by multiplying the recovery ratio (CFUs recovered from nasal wash or lung homogenate divided by CFUs used to infect mice intranasally) by 10^6^ (to simplify statistical comparisons and facilitate visual comparisons).

### Noncompetitive Growth of QRSP Mutants in Vivo

To establish noncompetitive pneumococcal infections with EF3030, Phe/Phe, and Phe/Tyr, 10^6^ CFUs were used for colonizations, and 10^7^ CFUs were used for lung infections, as described above. EF3030 was used to infect 39 mice (10 for colonization and 29 for pneumonia), Phe/Phe was used to infect 25 mice (5 for colonization and 20 for pneumonia), and Phe/Tyr was used to infect 24 mice (5 for colonization and 19 for pneumonia). Mice were killed after 7 days, and samples were collected and analyzed as described above.

### Statistical Analysis

The Wilcoxon matched-pairs signed-rank test was used to compare the numbers of generations for each competing pair in in vitro competitive growth experiments and to compare the PRUs in in vivo competitive infections. For noncompetitive infections, PRUs of EF3030, Phe/Phe, and Phe/Tyr were compared by using the Mann-Whitney unpaired 2-tailed test. Statistical tests were conducted with the InStat program (GraphPad Software, Inc., San Diego, CA, USA). A p value <0.05 was considered statistically significant.

## Results

### QRDR Mutations

The QRDR mutations in *gyrA* and *parC* of the clinical QRSP (donor strains CT01147 and UAB169), the mutants (Phe/Phe and Phe/Tyr), and parent strain (EF3030) were sequenced to confirm the presence of QRDR mutations and because genetic transformation has been associated with increased mutation frequency (21,22). The transformation fragment for *gyrA* consisted of 1,325 bp, of which 660 inclusive of the QRDR were sequenced. Likewise, the transformation fragment for *parC* consisted of 778 bp, of which 446 were sequenced. The *gyrA* and *parC* QRDR mutations in the mutants (Phe/Phe and Phe/Tyr) matched those of the corresponding donor strains (CT01147 and UAB169). Phe/Phe also contained 2 additional synonymous, nonquinolone resistance–conferring mutations in *gyrA* (data not shown). The levofloxacin MICs for the Phe/Phe and Phe/Tyr mutants were both 16 μg/mL, verifying the degree of resistance to the fluoroquinolone class of antimicrobial agents.

### Colonization Model

**Figure 1:**
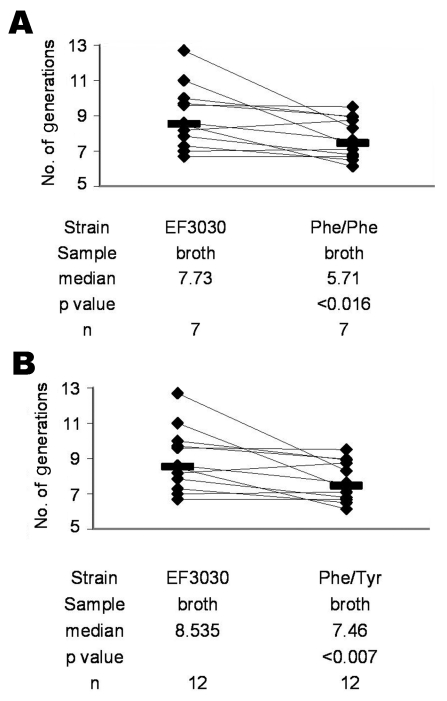
In vitro competition between *Streptococcus pneumoniae* EF3030 and the Phe/Phe mutant (A) and between EF3030 and the Phe/Tyr mutant (B) in liquid medium (broth). Bars indicate medians. Lines connect strains competing in the same broth. p values were calculated by the Wilcoxon matched-pairs signed-rank test.

**Figure 2:**
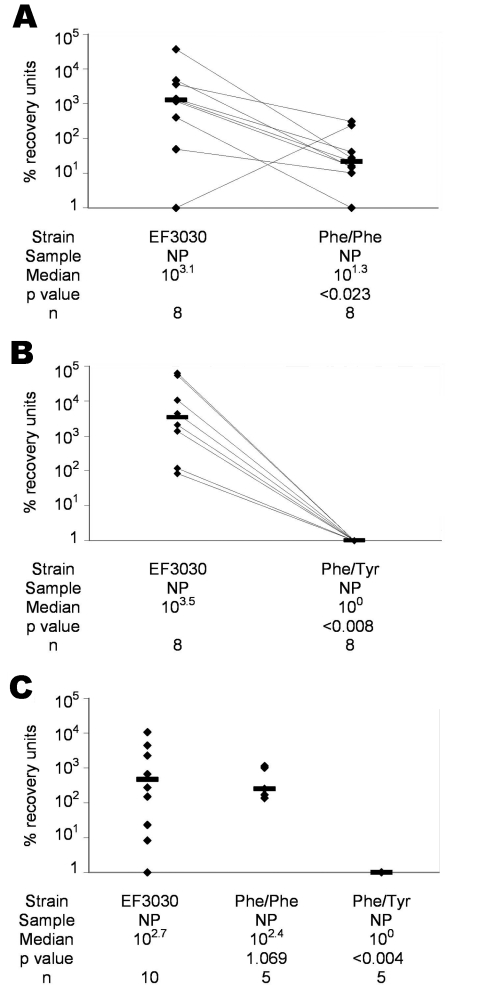
Percentage recovery units (PRUs: CFUs recovered from nasal wash or lung homogenate divided by CFUs originally used to infect mice and multiplied by 10^6^) for competitive colonization between *Streptococcus pneumoniae* EF3030 and the Phe/Phe mutant (A), EF3030 and the Phe/Tyr mutant (B), and noncompetitive colonization of EF3030, Phe/Phe, and Phe/Tyr (C). Lines connect data from the same mouse. Bars indicate median PRUs. NP, nasopharynx. p values were calculated by the Wilcoxon matched-pairs signed-rank test (A and B) and the Mann-Whitney unpaired 2-tailed test (C).

Overall, EF3030 underwent more generations per 6-hour in vitro growth period than either Phe/Phe (p<0.016) (Figure 1A) or Phe/Tyr (p<0.007) (Figure 1B). Of 10 mice intranasally infected with approximately equal amounts (10^6^ CFUs) of EF3030 and Phe/Phe, 8 were colonized. Among these 8 mice, EF3030 outcompeted Phe/Phe (p<0.023) (Figure 2A). Of 10 mice intranasally infected with approximately equal amounts of EF3030 and Phe/Tyr, 8 were colonized. Among these 8 mice, EF3030 outcompeted Phe/Tyr (p<0.008) (Figure 2B).

When mice were infected intranasally with 10^6^ CFUs of EF3030, Phe/Phe, or Phe/Tyr, Phe/Phe and EF3030 were recovered in similar numbers (p = 1.069), but Phe/Tyr was recovered in much lower numbers than EF3030 (p<0.004) (Figure 2C). Thus, although Phe/Phe was less efficient at nasopharyngeal colonization when competing with EF3030, it colonized as well as EF3030 when tested alone. Phe/Tyr was less efficient than EF3030 at colonizing, whether or not it was in direct competition with EF3030.

### Pneumonia Model

**Figure 3:**
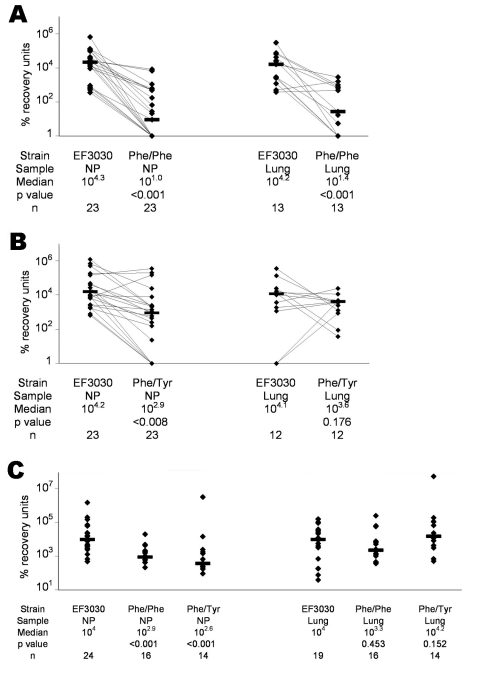
Percentage recovery units (PRUs: CFUs recovered from nasal wash or lung homogenate divided by CFUs originally used to infect mice and multiplied by 10^6^) for competitive pneumonia infection with *Streptococcus pneumoniae* EF3030 and the Phe/Phe mutant (A), EF3030 and the Phe/Tyr mutant (B), and noncompetitive pneumonia with EF3030, Phe/Phe, and Phe/Tyr (C). Lines connect data from the same mouse. Bars indicate median PRUs. NP, nasopharynx. p values were calculated by the Wilcoxon matched-pairs signed-rank test (A and B) and the Mann-Whitney unpaired 2-tailed test (C).

Of the 23 mice infected with approximately equal amounts (10^4^ CFUs of each strain or 10^6^ CFUs of each strain) of EF3030 and Phe/Phe, all 23 were colonized nasopharyngeally, and lung infection developed in 13 of 23. EF3030 outcompeted Phe/Phe in both the nasopharynx (p<0.001) and the lungs (p<0.001) (Figure 3A).

Of the 23 mice infected with approximately equal amounts (10^4^ CFUs or 10^6^ CFUs of each) of EF3030 and Phe/Tyr, all 23 were colonized nasopharyngeally, and lung infections developed in 12 of 23. We observed no significant difference in PRUs with the 2 different inocula. EF3030 outcompeted Phe/Tyr in the nasopharynx (p<0.008) but not in the lungs (p<0.176) (Figure 3B). Thus, when anesthetized mice were infected with both EF3030 and a mutant (Phe/Phe or Phe/Tyr), EF3030 outcompeted each mutant in the nasopharynx, but EF3030 outcompeted only Phe/Phe in the lungs.

Of the 29 mice monoinfected with 10^7^ CFUs of EF3030, 5 died of infection and 24 were colonized nasopharyngeally. Lung infections developed in 19 of these 24 (Figure 3C). Of the 20 mice monoinfected with 10^7^ CFUs of Phe/Phe, 4 died of infection and 16 were colonized nasopharyngeally; lung infections developed in all 16. Of the 19 mice monoinfected with 10^7^ CFUs of Phe/Tyr, 5 died of presumed pneumonia, and 14 were colonized nasopharyngeally; lung infections developed in all 14.

Among these monoinfections, EF3030 was recovered from the nasopharynx in quantities significantly different from those of Phe/Phe (p<0.001) and Phe/Tyr (p<0.001) (Figure 3C). In the lungs, however, EF3030 was not recovered in numbers significantly different those of from either Phe/Phe (p = 0.453) or Phe/Tyr (p = 0.152). Thus, even in the absence of competition, EF3030 was recovered in higher numbers than those of both mutants in the nasopharynx, but was not recovered in higher numbers than those of either mutant in the lungs.

## Discussion

Although fluoroquinolone resistance in *S. pneumoniae* remains very low in North America, it has begun to increase in recent years (15,23) and is especially high in some Asian countries that already have high β-lactam and macrolide resistance rates (24). Pneumococcal resistance to fluoroquinolones is largely mediated by de novo point mutations in the *gyrA* and *parC* genes encoding DNA gyrase and topoisomerase, respectively, in the QRDRs (25). A specific single-point mutation in either of these genes confers low-level resistance, with high-level resistance generally requiring a point mutation in both *gyrA* and *parC* QRDRs. QRSP are generally clonally unrelated, although there have been some reports of clonal dissemination, and fluoroquinolone resistance has now been reported in several international clones (10–13,26).

The fitness of pathogenic bacteria to cause disease relies on several factors, including colonization of the host, evasion of host defenses, propagation on or inside the host, and transmission to a new host. Antimicrobial resistance can be associated with a decrease in bacterial fitness (3,27). A measure of fitness of antimicrobial-resistant pathogens could aid in the prediction of the future rates of disease caused by these bacteria, guide recommendations for empiric therapy for some bacterial infections, and direct the development of new antimicrobial drugs. Although other studies have investigated the fitness of antimicrobial-resistant pathogens including *S. pneumoniae* (6,7), the focus has frequently been on resistance to β-lactam antimicrobial drugs, and only a few have investigated the relative fitness of QRSP (4,5).

In our current study, we sought to investigate the fitness of QRSP mutants. We postulated that QRSP may have reduced fitness because fluoroquinolone resistance rates remain very low, and naturally occurring QRSP isolates are generally clonally unrelated. When in competition with EF3030, the Phe/Phe mutant, which contains the GyrA: Ser81Phe and ParC: Ser79Phe mutation combination often found in clinical QRSP (11,18,24,28–30), was inferior in all 3 models tested. However, in the absence of competition with EF3030, Phe/Phe was only inferior in nasopharyngeal colonization but was as able as EF3030 to produce lung infection. Conversely, the Phe/Tyr mutant, which contains the GyrA: Ser81Phe and ParC: Ser79Tyr mutation combination found more often in laboratory-selected mutants than in clinical QRSP (18,24,28–30), was inferior in vitro and in nasopharyngeal colonization but was as able as EF3030 to produce lung infection, regardless of competition from EF3030. Though counterintuitive, this probably occurred because of the nature of the lung infection model, in which bacteria are intranasally instilled into anesthetized mice without the prerequisite for nasopharyngeal colonization. In fact, nasopharyngeal colonization resulting from the lung infection model is more the result of retrograde movement of bacteria from the lungs to the nasopharynx than of initial collection of bacteria in the nasopharynx when first infected. Why QRDR mutations tended to confer more fitness costs in the nasopharyngeal mucosa than in the lungs is not clear, but it is possible that commensal bacteria may have provided more competition in the nasopharynx than in the lungs, and therefore the mutants displayed greater fitness reductions when in competition with both wild-type *S. pneumoniae* and commensal bacteria. Alternatively, phase variation in pneumococcal opacity may play a role in the difference in fitness of the mutants in the lung versus the nasopharynx, since the opaque phase tends to predominate in invasion, and the transparent phase predominates in colonization (31). These 2 phases express very different complements of virulence factors, which suggest that the processes involved in bacterial survival in these 2 niches can be very different.

In mice that had been colonized and in those with lung infections, fewer organisms were recovered than were infected, i.e., no bacterial growth was detectable in the animals. It may be postulated that these models are simply measuring the relative death rates of the Phe/Phe and Phe/Tyr mutants, as compared to EF3030, and not actual survival and growth. In a study by Balachandran et al. (32), evidence was presented indicating that pneumococci multiply during colonization. To our knowledge, no studies have investigated pneumococcal turnover in the lung, but since the lungs of CBA/N mice contain many neutrophils (33), the bacteria would likely have to multiply to compensate for being killed, based on the number of bacteria recovered from the lungs.

Although several studies have investigated the fitness of antimicrobial-resistant pathogens (8,34,35) and antimicrobial-resistant *S. pneumoniae* (6,7,36,37), few have investigated the fitness of fluoroquinolone-resistant bacteria (4,5). Our results are in contrast to those of Gillespie et al. (4), who found a significant decrease in the relative fitness of Tyr/Tyr, but not of Phe/Tyr, compared to wildtype, in in vitro growth experiments with *S. pneumoniae*. Conversely, our results are supported by Giraud et al. (8), who reported a decrease in relative fitness in high-level fluoroquinolone-resistant *Salmonella enterica* serovar Typhimurium in in vitro growth and chicken gut colonization experiments, and by Azoulay-Dupuis et al. (5), who demonstrated that clinical strains of QRSP were less virulent in outbred mice than a quinolone-susceptible laboratory strain and its quinolone-resistant isogenic mutant.

If QRSP are less efficient than fluoroquinolone-susceptible *S. pneumoniae* at colonizing humans, the result could explain the few reports of clonal lineages of QRSP. Nasopharyngeal colonization precedes pneumonia and is the reservoir from which person-to-person transmission occurs (38). Therefore, a pneumococcus that is inefficient in colonizing the nasopharynx would be less efficient in producing lung infection, no matter how efficiently the organism infects the lungs; likewise, this organism would be less likely to disseminate clonally in the community. However, the fact that lung infection is not attenuated by fluoroquinolone resistance indicates that the resistant strains selected in patients by antimicrobial treatment may still cause severe disease, and possibly death, as has been reported (39,40).

We have attempted to measure the relative fitness of the 2 most commonly occurring QRDR mutation combinations. While different mutations may have different affects on fitness, we found that strains containing these common QRDR mutations appeared to have reduced fitness in the absence of antimicrobial drugs both in vitro and in vivo. Thus, QRSP may have reduced ability to initiate infections in the absence of fluoroquinolone selection and may be inefficient at displacing resident susceptible strains and therefore causing disease. This suggests that the judicious use of antimicrobial drugs may keep the prevalence of QRDR clones low because of their relatively low fitness.

The few reports of clonal spread of QRSP and of fluoroquinolone resistance in multidrug-resistant isolates raise the possibility that these isolates may have already acquired compensatory mutations. Continued surveillance is very important in understanding the epidemiology of QRSP. Overall, fluoroquinolone resistance rates remain very low, most resistance arises in genetically diverse strains, and clonal dissemination is likely still not a major contributor to the appearance of QRSP.
